# Complete dataset for 2-treatment, 2-sequence, 2-period efavirenz bioequivalence study conducted with nightly dosing

**DOI:** 10.1016/j.dib.2016.03.036

**Published:** 2016-03-15

**Authors:** Manuel Ibarra, Laura Magallanes, Marianela Lorier, Marta Vázquez, Pietro Fagiolino

**Affiliations:** Bioavailability and Bioequivalence Centre for Medicine Evaluation (CEBIOBE) – Faculty of Chemistry, Universidad de la República, Montevideo, Uruguay

**Keywords:** Efavirenz, Bioequivalence

## Abstract

The efavirenz pharmacokinetic raw data presented in this article was obtained in an average bioequivalence study between a local brand and Stocrin (Merck Sharp & Dohme, purchased from Australia, batch H009175, expiration date November 2013). Dose was administered at night (9:00 p.m.) two hours after food intake. Fourteen healthy subjects, 8 women and 6 men, completed the study. For each subject, 15 data points until 96 h post-administration are included. Subject demographic characteristics and sequences of administration are provided along with individual pharmacokinetic profiles of efavirenz obtained for both formulations after a single oral dose of 600 mg. This data provides information in support of the research article “Sex-by-formulation interaction assessed through a bioequivalence study of efavirenz tablets” [1].

**Specifications Table**

**Table:** 

Subject area	*Pharmaceutical Sciences*
More specific subject area	*Bioavailability and Bioequivalence*
Type of data	*Table*
How data was acquired	*High performance liquid chromatography with ultraviolet detection*
Data format	*Raw*
Experimental factors	*Blood plasma samples were processed for efavirenz extraction and quantification as described elsewhere*[1]
Experimental features	*A 2-treatment, 2-period, 2-sequence, randomized and balanced crossover design was carried out with 14 healthy subjects, 8 women and 6 men*
Data source location	*Montevideo, Uruguay*
Data accessibility	*Data supplied with this article*

**Value of the data**

• Efavirenz pharmacokinetic data with intensive sampling obtained after nightly dosing. Characterization of efavirenz pharmacokinetics after single oral nightly dose can be performed with this data. Efavirenz chronopharmacokinetics and evaluation of daytime administration on bioequivalence can be studied by comparison with standard bioequivalence trials.
• Comparison of Reference (Stocrin®, Merck Sharp & Dohme) performance in different populations. Data collected for the Reference formulation could be managed with the objective of comparing its performance in different populations.
• Development of in vitro–in vivo correlations. Efavirenz is classified as Class II drug in the Biopharmaceutical Classification System (BCS), its oral absorption is limited by its dissolution. Appropriate in vitro assays could help to explain and predict dosage bioavailability.

## 1 Data

In this article, raw pharmacokinetic data obtained in an efavirenz average bioequivalence study carried out on 14 healthy subjects is presented. Venous plasma efavirenz concentrations were obtained after a 600 mg oral dose of a local brand (Test) and the Reference, Stocrin (Merck Sharp & Dohme).

## 2 Experimental design, materials and methods

A randomized two-treatment, two-period, two-sequence, single-dose crossover study with a washout period of 28 days was performed for bioequivalence evaluation of Test formulation. Sixteen healthy Caucasian subjects were initially recruited, 8 males and 8 females. Dose (600 mg) was administered at night (9:00 p.m.) two hours after the intake of a standardized dinner. Blood samples were obtained up to 96 h post-dosing. Efavirenz concentrations were measured in venous plasma by high performance liquid chromatography with UV detection at 205 nm (HPLC-UV), using a validated analytical method with a lower limit of quantification of 50 ng/mL. Detailed information regarding this method was previously described [1]. The study protocol followed the tenets of the Declaration of Helsinki adopted by the World Medical Association in 1964 and its successive amendments, being previously approved by the Institutional Ethics Review Committee of the Faculty of Chemistry – Universidad de la República. All volunteers received a leaflet with study details and efavirenz information and signed a consent form before their entry to the study. Table 1 summarizes the demographic characteristics of participating volunteers, along with the assigned sequence of product administration. Fourteen volunteers finished the study. Subject 16 did not show up the first day of the study. Subject 11 presented rash after administration of Test formulation. Fortunately, both individuals belonged to different sequence of administration, and so replacement was not necessary to maintain the crossover balanced.

**Fig. 1:**
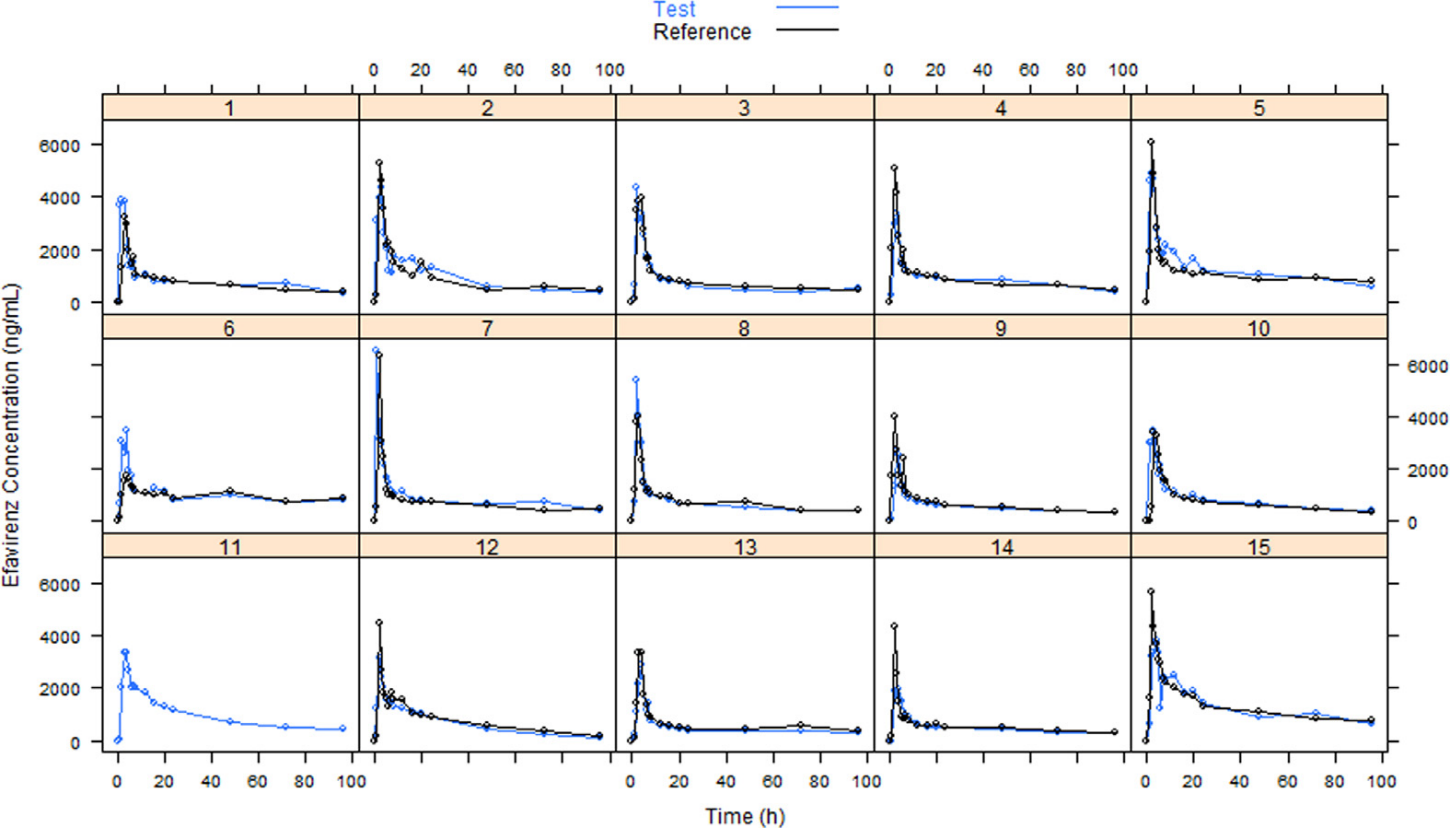
Individual efavirenz plasma concentration–time profiles after the administration of Test and Reference formulations.

**Fig. 2:**
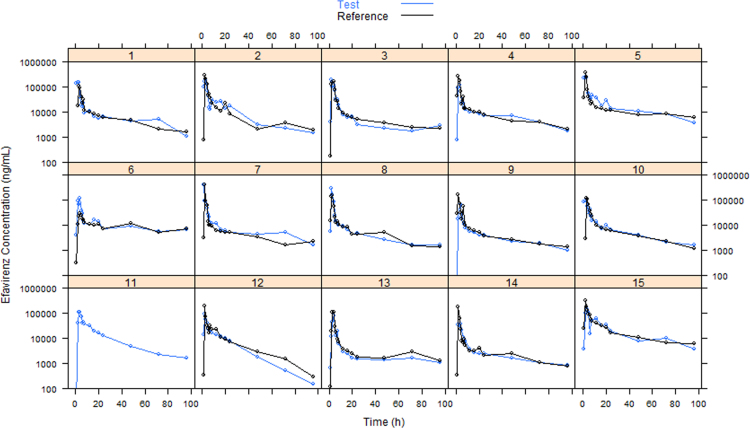
Individual efavirenz plasma concentration–time profiles (log-linear scale) after the administration of Test and Reference formulations.

Pharmacokinetic data of efavirenz after single oral dose of Test and Reference is presented in Fig. 1, Fig. 2. For subject 11, only Test data is available. Individual efavirenz plasma concentration from both formulations could be retrieved from a previously published article [2].

## Figures and Tables

**Table 1:** Subject demographic characteristics.

**Subject IDS**	**Sex**	**Weight (kg)**	**Age (years)**	**Height (cm)**	**Smoker status**^a^	**Sequence of administration**^b^
**1**	Female	66	49	165	NS	RT
**2**	Female	70	37	170	NS	RT
**3**	Female	70	20	164	NS	TR
**4**	Female	56	19	160	NS	TR
**5**	Female	61	20	168	NS	TR
**6**	Female	60	20	160	S	RT
**7**	Female	63	26	161	NS	RT
**8**	Female	65	35	174	S	TR
**9**	Male	85	35	184	S	RT
**10**	Male	93	28	181	NS	RT
**11**	Male	74	23	181	NS	TR
**12**	Male	76	20	174	NS	TR
**13**	Male	106	46	167	S	TR
**14**	Male	110	22	181	NS	RT
**15**	Male	74	18	173	NS	TR
**16**	Male	70	49	165	NS	RT

a NS: non-smoker; S: smoker.

b TR: Test-Reference; RT: Reference-Test.
